# Association of Food Insecurity and Sarcopenia: The National Health and Nutrition Examination Surveys

**DOI:** 10.1093/geroni/igaa057.2793

**Published:** 2020-12-16

**Authors:** John Batsis, Curtis Petersen, Tyler Gooding

**Affiliations:** 1 Dartmouth College, Lebanon, New Hampshire, United States; 2 Dartmouth Hitchcock Medical Center, Lebanon, New Hampshire, United States

## Abstract

Understanding the association between food insecurity and sarcopenia can inform policies that address healthcare disparities. We identified 2,965 subjects aged ≥60 years with grip strength and food security data from the National Health and Nutrition Examination Surveys 2011-14. Sarcopenia was defined using grip strength <26kg for men and <16kg for women, and food security with the 18-item US Household Food Security Survey. Logistic regression evaluated the association of food insecurity (referent = full security) with sarcopenia, adjusting for age, sex, marital status, race/ethnicity, education, body mass index, smoking, and comorbidities. Mean age (% female) was 76.6 (60.9%) and 68.9 years (53.4%) with and without sarcopenia, respectively. Sarcopenia prevalence was 8.4%. Rates of full food security were higher in those without sarcopenia (90% vs. 84.2%;p<0.001). Food insecurity was strongly associated with sarcopenia (OR 1.79 [1.18, 2.72]), suggesting a need for both longitudinal and interventional studies to target these disparities.

